# 新药时代原发性浆细胞白血病预后因素分析

**DOI:** 10.3760/cma.j.cn121090-20240129-00042

**Published:** 2024-07

**Authors:** 晶晶 邓, 小云 金, 之尧 张, 慧星 周, 光忠 杨, 传营 耿, 原 菅, 文明 陈, 文 高

**Affiliations:** 首都医科大学附属北京朝阳医院，北京 100020 Beijing Chao-Yang Hospital, Capital Medical University, Beijing 100020, China

**Keywords:** 白血病，浆细胞, 预后, 高钙血症, 维持治疗, 治疗结果, Leukemia, plasma cell, Prognosis, Hypercalcemia, Maintenance therapy, Treatment outcome

## Abstract

**目的:**

探讨新药时代原发性浆细胞白血病（pPCL）的预后因素。

**方法:**

回顾性收集2011年至2022年就诊于首都医科大学附属北京朝阳医院血液科66例pPCL患者的临床资料，分析其预后因素。

**结果:**

66 例pPCL患者中位发病年龄为59（29～79）岁。中位总生存（OS）期为19.0（95％*CI* 10.4～27.6）个月，中位无进展生存（PFS）期为11.0（95％*CI* 6.5～15.6）个月。治疗后最佳疗效≥非常好的部分缓解（VGPR）患者的中位OS期和PFS期均显著长于疗效≤部分缓解（PR）患者（中位OS期：33.0个月对6.0个月，*P*<0.001；中位PFS期：16.0个月对3.0个月，*P*<0.001）。接受自体造血干细胞移植患者的OS期较未接受患者显著延长（49.0个月对6.0个月，*P*＝0.002），PFS期也有延长趋势（19.0个月对8.0个月，*P*＝0.299）。接受维持治疗患者的中位OS期、PFS期较未接受维持治疗患者显著延长（中位OS期：56.0个月对4.0个月，*P*<0.001；中位PFS期：20.0个月对2.0个月，*P*<0.001）。多因素分析结果表明，高钙血症是影响pPCL患者OS的独立危险因素（*HR*＝3.204，95％*CI* 1.068～9.610，*P*＝0.038）；接受维持治疗（*HR*＝0.075，95％*CI* 0.022～0.253，*P*<0.001）、治疗后疗效≥VGPR（*HR*＝0.175，95％*CI* 0.048～0.638，*P*＝0.008）是影响pPCL患者OS的独立保护因素。

**结论:**

在新药时代，高钙血症、接受维持治疗、治疗后疗效≥VGPR是pPCL的独立预后因素。

## Abstract (en)

**Objective:**

To explore the prognostic factors of primary plasma cell leukemia（pPCL）in the era of novel agents.

**Methods:**

The clinical data of 66 patients with pPCL treated at the Department of Haematology, Beijing Chao-Yang Hospital, Capital Medical University from 2011 to 2022 were retrospectively collected to analyze their prognostic factors.

**Results:**

Among the 66 patients with pPCL, the median age was 59（range: 29–79）years. The median overall survival（OS）duration was 19.0（95% *CI* 10.4–27.6）months, and the median progression-free survival（PFS）duration was 11.0（95% *CI* 6.5–15.6）months. The median OS and PFS were significantly longer in patients with the best post-treatment response of very good partial remission（VGPR）or better than in patients with a response of partial remission（PR）or worse（median OS: 33.0 months *vs* 6.0 months, *P*<0.001; median PFS: 16.0 months *vs* 3.0 months, *P*<0.001）. OS was significantly longer in patients who underwent autologous hematopoietic stem cell transplantation than in those who did not undergo transplantation（49.0 months *vs* 6.0 months, *P*＝0.002）, and there was a trend toward a longer PFS in patients who underwent transplantation than in those who did not undergo transplantation（19.0 months *vs* 8.0 months, *P*＝0.299）. The median OS and PFS were significantly longer in patients who received maintenance therapy than in those who did not receive maintenance therapy（median OS: 56.0 months *vs* 4.0 months, *P*<0.001; median PFS: 20.0 months *vs* 2.0 months, *P*<0.001）. Multivariate analysis showed that hypercalcemia was an independent risk factor（*HR*＝3.204, 95% *CI* 1.068–9.610, *P*＝0.038）for patients with pPCL, while receiving maintenance therapy（*HR*＝0.075, 95% *CI* 0.022–0.253, *P*<0.001）and post-treatment response of VGPR or better（*HR*＝0.175, 95% *CI* 0.048–0.638, *P*＝0.008）were independent protective factors for patients with pPCL.

**Conclusion:**

In the era of novel agents, hypercalcemia, receiving maintenance therapy, and post-treatment response of VGPR or better are independent prognostic factors for pPCL.

原发性浆细胞白血病（pPCL）是一种罕见的恶性浆细胞疾病，其临床病程呈侵袭性，预后极差。传统的浆细胞白血病（PCL）诊断标准定义为外周血的循环浆细胞（CPC）比例≥20％和（或）CPC绝对计数≥2×10^9^/L[1]。然而，近年的研究表明，CPC比例≥5％的多发性骨髓瘤患者的预后与传统定义的pPCL患者相似[2]–[3]。因此，国际骨髓瘤工作组（IMWG）在2021年将PCL的诊断标准修订为外周血涂片中CPC比例≥5％[4]。

在传统化疗时代，pPCL患者的生存期仅为6～10个月[5]。而新药时代，蛋白酶体抑制剂（PIs）、免疫调节剂（IMiDs）、CD38单抗等新药的临床应用及联合造血干细胞移植（HSCT）增加了pPCL患者的缓解深度，并延长了患者的生存期[6]–[8]，但其整体生存仍很差。近年的研究表明，pPCL患者的生存与缓解深度、细胞遗传学、肿瘤负荷及治疗方式等相关。然而，由于不同研究患者的选取及治疗方案存在较大异质性，很难建立相对一致的预后模型。本研究对首都医科大学附属北京朝阳医院血液科收治的pPCL连续性病例进行分析，探讨新药时代下符合新诊断标准的pPCL患者的生存情况及预后影响因素。

## 病例与方法

1. 病例：根据2021年pPCL IMWG诊断标准[4]，回顾性收集2011年8月至2022年10月就诊于首都医科大学附属北京朝阳医院血液科的66例pPCL患者的临床资料。本研究经首都医科大学附属北京朝阳医院医学研究伦理委员会批准（伦理批号：2024-科-12）。

2. 间期FISH检测常见遗传学异常：使用磁珠分离、富集骨髓中CD138阳性细胞。探针包括IGH/FGFR3、IGH/MAF、IGH/CCND1、1q21、p53/CEP17等，观察200个分裂间期细胞荧光杂交信号。按照欧洲骨髓瘤工作组设定的阈值标准，del（17p）和1q21+的阳性阈值为≥20％，基因融合的阳性阈值为≥10％。

3. 疗效评估标准：pPCL的疗效评估参考2013年版IMWG疗效评估标准，包括严格意义的完全缓解（sCR）、完全缓解（CR）、非常好的部分缓解（VGPR）、部分缓解（PR）、疾病稳定（SD）和疾病进展（PD）[1]。

4. 随访：随访截止时间为2023年3月，中位随访时间为46.0（95％*CI* 19.7～72.3）个月。总生存（OS）期定义为患者自首次确诊至因任何原因死亡或随访截止的时间，无进展生存（PFS）期定义为患者自首次确诊到疾病复发或进展、因任何原因死亡或随访截止的时间。

5. 统计学处理：采用SPSS 25.0软件进行统计学分析。计数资料用例数（百分比）表示，计量资料用中位数（范围）表示，应用卡方检验及Fisher精确检验进行差异性比较。采用Kaplan-Meier法进行生存分析，Log-rank检验进行差异性分析。多因素分析采用Cox风险回归模型，*P*<0.05为差异有统计学意义。

## 结果

1. 基线特征：66例pPCL患者的中位发病年龄为59（29～79）岁，其中男性35例。CPC比例为5％～19％的患者47例（71.2％），中位CPC比例为11.5％（5％～86％），中位骨髓浆细胞比例为66.25％（12.5％～96.5％）。免疫分型结果显示，轻链型29例（43.9％），IgG型26例（39.4％），IgA型7例（10.6％），IgD型3例（4.5％），非分泌型1例（1.5％）。伴高危细胞遗传学异常［FISH检出del（17p）、t（4;14）、t（14;16）］的患者占42.6％（26/61），伴t（11;14）的患者占41.8％（23/55），伴1q21+的患者占56.7％（34/60）。38例患者进行了核型分析，其中复杂核型19例。28例（46.7％）患者伴LDH升高（>250 U/L），19例（30.6％）患者伴高钙血症（校正血清钙>2.75 mmol/L），57例（87.7％）患者伴贫血（HGB<100 g/L），27例（42.2％）患者伴血小板减少（PLT<100×10^9^/L），24例（36.4％）患者伴肾功能不全（血肌酐≥177 µmol/L）。

2. 治疗方案：3例患者在诊断后未进行原发病治疗，其他患者均接受了新药诱导治疗，其中7例（11.1％）患者接受了含CD38单抗方案的诱导治疗，22例（34.9％）患者接受了以PIs为基础的治疗，4例（6.3％）患者接受以IMiDs为基础的化疗，37例（58.7％）患者接受PIs联合IMiDs治疗。19例（30.2％）患者在接受诱导治疗后进行了自体造血干细胞移植（auto-HSCT），1例（1.6％）患者接受了异基因造血干细胞移植（allo-HSCT），46例患者未接受HSCT治疗。6例患者失访，维持治疗方案不详，在进一步分析中被剔除，35例患者未进行维持治疗，25例患者进行了维持治疗，其中18例患者接受IMiDs维持治疗，4例患者接受PIs维持治疗，3例患者接受了PIs联合IMiDs维持治疗。

3. 疗效：19例患者因早期死亡或失访未行疗效评估。47例患者进行了规范的疗效评估，最佳疗效达CR率、≥VGPR率分别为38.3％（18/47）及72.3％（34/47）。接受auto-HSCT患者的CR率、≥VGPR率分别为66.7％（12/18）及100％（18/18），未接受auto-HSCT患者的CR率、≥VGPR率分别为23.1％（6/26）及61.5％（16/26），两组的差异均有统计学意义（CR率：*χ*^2^＝8.360，*P*＝0.004；≥VGPR率：*χ*^2^＝8.959，*P*＝0.003）。接受维持治疗患者的CR率、≥VGPR率分别为73.9％（17/23）及95.7％（22/23），未接受维持治疗患者的CR率、≥VGPR率分别为4.2％（1/24）及50％（12/24），两组的差异有统计学意义（CR率：*χ*^2^＝24.177，*P*<0.001；≥VGPR率：*χ*^2^＝12.233，*P*<0.001）。27例疗效≥VGPR的患者进行了微小残留病（MRD）检测，其中8例患者二代流式细胞术MRD阴性（<1×10^−5^）。

4. 生存分析：截至末次随访，共43例（65.2％）患者死亡，其中OS期≤3个月的患者15例。总体的中位OS期为19.00（95％*CI* 10.36～27.64）个月，中位PFS期为11.00（95％*CI* 6.46～15.55）个月。CPC比例5％～19％与CPC比例≥20％的患者中位OS期分别为21.00（95％*CI* 12.93～29.07）个月、19.00（95％*CI* 0～42.25）个月，中位PFS期分别为11.00（95％*CI* 4.28～17.72）个月、9.00（95％*CI* 0～24.40）个月，差异均无统计学意义（*P*值分别为0.207、0.173）（图1）。根据患者治疗后的最佳疗效，获得≥VGPR及≤PR的患者中位OS期分别为33.00（95％*CI* 11.84～54.17）个月及6.00（95％*CI* 2.48～9.52）个月，中位PFS期分别为16.00（95％*CI* 7.99～24.01）个月及3.00（95％*CI* 2.22～3.78）个月，差异均有统计学意义（*P*值均<0.001）（图2）。接受维持治疗患者的中位OS期［56.00（95％*CI* 36.05～75.95）个月对4.00（95％*CI* 1.32～6.68）个月］及中位PFS期［20.00（95％*CI* 14.70～25.30）个月对2.00（95％*CI* 1.38～2.62）个月］较未接受维持治疗的患者显著延长（*P*值均<0.001）（图3）。接受及未接受auto-HSCT的患者中位OS期分别为49.00（95％*CI* 27.00～71.00）个月及6.00（95％*CI* 1.36～10.64）个月，差异有统计学意义（*P*＝0.002）；中位PFS期分别为19.00（95％*CI* 13.13～24.87）个月及8.00（95％*CI* 5.39～10.61）个月，差异无统计学意义（*P*＝0.299）（图4）。

**图1:**
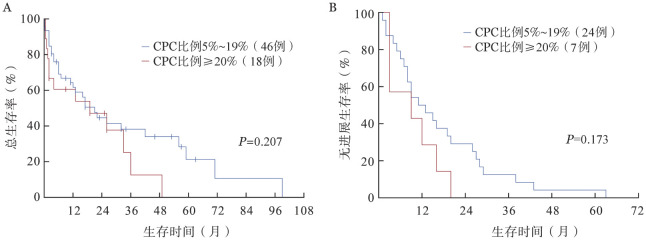
循环浆细胞（CPC）比例5％～19％与≥20％患者的总生存（A）和无进展生存（B）曲线

**图2:**
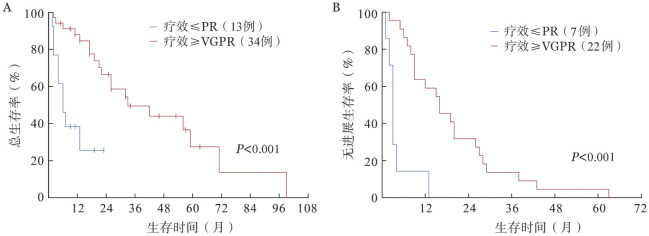
疗效≥非常好的部分缓解（VGPR）与≤部分缓解（PR）患者的总生存（A）和无进展生存（B）曲线

**图3:**
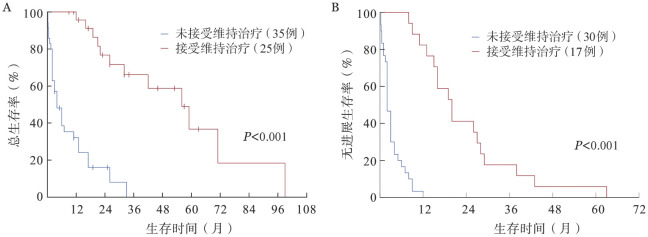
接受与未接受维持治疗患者的总生存（A）和无进展生存（B）曲线

**图4:**
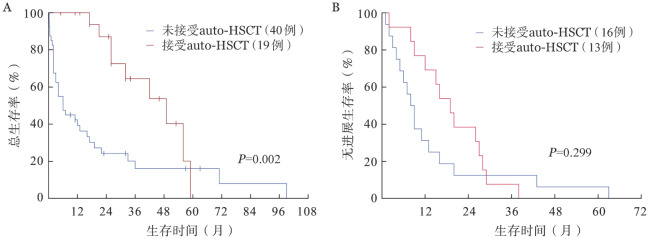
接受与未接受自体造血干细胞移植（auto-HSCT）患者的总生存（A）和无进展生存（B）曲线

5. 预后影响因素：将性别、年龄、外周血浆细胞比例、高危FISH异常、β_2_-微球蛋白、初诊时LDH、血钙、HGB、PLT、肌酐、是否接受auto-HSCT和维持治疗、治疗后最佳疗效等因素纳入单因素分析，进一步将*P*<0.05的因素纳入多因素分析。结果显示，高钙血症［*HR*＝3.204（95％*CI* 1.068～9.610），*P*＝0.038］、接受维持治疗［*HR*＝0.075（95％*CI* 0.022～0.253），*P*<0.001］及治疗后疗效≥VGPR［*HR*＝0.175（95％*CI* 0.048～0.638），*P*＝0.008］是pPCL患者的独立预后因素（表1）。

**表1:** 影响原发性浆细胞白血病患者总生存的单因素及多因素分析

因素	单因素分析		多因素分析	
	*HR*（95%*CI*）	*P*值	*HR*（95%*CI*）	*P*值
年龄（≥60岁，<60岁）	1.702（0.925～3.133）	0.088		
性别（男，女）	1.206（0.654～2.222）	0.549		
CPC比例（≥20%，<20%）	1.524（0.780～2.978）	0.218		
del(17p)（是，否）	2.140（0.872～5.251）	0.097		
t(4;14)（是，否）	1.634（0.821～3.252）	0.162		
t(14;16)（是，否）	0.732（0.223～2.407）	0.608		
β_2_-微球蛋白（>5.5 mg/L，≤5.5 mg/L）	1.616（0.731～3.573）	0.236		
LDH升高（>250 U/L，≤250 U/L）	0.888（0.465～1.695）	0.719		
高钙血症（校正血清钙>2.75 mmol/L，≤2.75 mmol/L）	2.367（1.214～4.614）	0.011	3.204（1.068～9.610）	0.038
HGB（<100 g/L，≥100 g/L）	0.818（0.341～1.960）	0.652		
PLT（<100×10^9^/L，≥100×10^9^/L）	0.781（0.417～1.462）	0.439		
肌酐升高（≥177 µmol/L，<177 µmol/L）	1.257（0.646～2.447）	0.501		
接受auto-HSCT（是，否）	0.330（0.155～0.701）	0.004	1.407（0.462～4.279）	0.548
接受维持治疗（是，否）	0.106（0.044～0.255）	<0.001	0.075（0.022～0.253）	<0.001
疗效≥VGPR（是，否）	0.202（0.078～0.521）	0.001	0.175（0.048～0.638）	0.008

**注** CPC：循环浆细胞；auto-HSCT：自体造血干细胞移植；VGPR：非常好的部分缓解

## 讨论

本研究的基线特征显示，pPCL患者的肿瘤负荷高，伴高危细胞遗传学异常的比例也较高。即使接受新药及auto-HSCT治疗，其预后仍较差。CPC比例为5％～19％的患者与CPC比例≥20％的患者预后类似，中位OS及PFS的差异均无统计学意义。进一步分析显示，高钙血症、疗效≥VGPR及接受维持治疗均是pPCL的独立预后因素。

日本的一项研究分析了2005–2015年纳入的26例pPCL患者，42％的患者接受新药治疗，58％的患者接受传统化疗或未接受治疗，多因素分析表明，高钙血症是唯一的独立预后不良因素[9]。国际血液和骨髓移植研究中心（CIBMTR）2008–2015年的数据显示，移植前疗效≥VGPR的患者生存率更高且复发率更低[10]。希腊骨髓瘤工作组的一项研究分析新药时代真实世界pPCL患者的预后，纳入的50例患者中80％接受基于一种新药（PIs/IMiDs）的诱导治疗，结果表明，疗效≥VGPR是OS的独立预后因素[6]。近期一项拉丁美洲的多中心研究在2012–2020年收治了72例pPCL患者，维持治疗是OS的独立预后因素[11]。其他相关研究也表明，维持治疗可以显著延长患者的PFS期，且具有延长OS期的趋势[12]–[13]。上述研究对象均为符合传统诊断标准的pPCL患者，并未纳入CPC比例5％～19％的患者，且患者的诱导治疗方案存在很强的异质性，接受传统化疗的患者占较高比例（18％～47％）[6],[9]–[11]。本研究中，95.5％（63/66）的患者接受了基于新药的诱导治疗，半数以上患者接受了PIs联合IMiDs治疗，因此更能代表新药时代符合修订标准的pPCL患者的特征，具有更现实的指导意义。

希腊骨髓瘤工作组研究结果表明，VRd方案（硼替佐米+来那度胺+地塞米松）或基于CD38单抗的四药疗法、del（17p）阳性、PLT<100×10^9^/L是影响pPCL患者OS的独立预后因素[14]。韩国骨髓瘤工作组发现，存在浆细胞瘤、β_2_-微球蛋白升高预示OS较差[15]。中国医学科学院血液病研究所对158例pPCL患者进行了生存分析，结果表明，LDH升高是唯一的独立预后因素[16]。国外一项多中心回顾性研究对2006–2016年间纳入的117例pPCL患者进行了预后分析，结果表明，患者的生存结局与年龄≥60岁、PLT≤100×10^9^/L和外周血浆细胞数≥20×10^9^/L相关[8]。Winship癌症研究所对38例pPCL患者进行了生存及预后分析，发现疗效≥CR为生存的独立预后因素[12]。梅奥中心的研究报道显示，最佳疗效为CR和无高危细胞遗传学异常是OS的预后因素[17]。在本研究中，虽然具有高危细胞遗传学异常患者的中位OS时间较标危患者缩短（19个月对22个月），但差异无统计学意义（*P*＝0.232）。中国医学科学院血液病研究所的一项研究也表明，高危细胞遗传学异常对pPCL预后的影响不如多发性骨髓瘤大[16]，可能与pPCL中位OS期太短有关。除了应用含CD38单抗治疗方案及伴浆细胞瘤患者数量太少无法统计外，本研究对上述其他预后因素均进行了研究，未发现差异有统计学意义。

最近有报道显示，伴t（11;14）的pPCL患者的OS期明显较不伴t（11;14）患者延长[18]，且t（11;14）可作为一种重要的生物标志物预测小分子Bcl-2抑制剂Venetoclax的敏感性[19]。但本研究的分析结果表明，伴t（11;14）患者的中位OS与不伴此易位患者相比差异无统计学意义，可能与本研究患者数量较少，存在统计学偏倚相关。除此之外，Karnofsky功能状态评分>90分[10]、美国东部肿瘤协作组评分>2[20]也被发现与生存相关。由于上述多项研究发表时诊断标准尚未更新，只有少数研究以5％作为pPCL诊断界值，因此本研究的结果在新药时代新诊断标准下具有一定意义。

综上所述，pPCL进展迅速，预后差，整体生存的改善仍然有限。本研究发现高钙血症、接受维持治疗、治疗后疗效≥VGPR是pPCL的独立预后因素。但由于本研究样本量较小，且为单中心回顾性研究，因此该结论是否具有普遍性还需更多前瞻性大样本研究进一步证实。
